# Placebo Response of Non-Pharmacological and Pharmacological Trials in Major Depression: A Systematic Review and Meta-Analysis

**DOI:** 10.1371/journal.pone.0004824

**Published:** 2009-03-18

**Authors:** André Russowsky Brunoni, Mariana Lopes, Ted J. Kaptchuk, Felipe Fregni

**Affiliations:** 1 Berenson-Allen Center for Noninvasive Brain Stimulation, Beth Israel Deaconess Medical Center, Harvard Medical School, Boston, Massachusetts, United States of America; 2 Department and Institute of Psychiatry, University of Sao Paulo, Sao Paulo, Brazil; 3 Osher Research Center, Harvard Medical School, Boston, Massachusetts, United States of America; Chiba University Center for Forensic Mental Health, Japan

## Abstract

**Background:**

Although meta-analyses have shown that placebo responses are large in Major Depressive Disorder (MDD) trials; the placebo response of devices such as repetitive transcranial magnetic stimulation (rTMS) has not been systematically assessed. We proposed to assess placebo responses in two categories of MDD trials: pharmacological (antidepressant drugs) and non-pharmacological (device- rTMS) trials.

**Methodology/Principal Findings:**

We performed a systematic review and meta-analysis of the literature from April 2002 to April 2008, searching MEDLINE, Cochrane, Scielo and CRISP electronic databases and reference lists from retrieved studies and conference abstracts. We used the keywords placebo and depression and escitalopram for pharmacological studies; and transcranial magnetic stimulation and depression and sham for non-pharmacological studies. All randomized, double-blinded, placebo-controlled, parallel articles on major depressive disorder were included. Forty-one studies met our inclusion criteria - 29 in the rTMS arm and 12 in the escitalopram arm. We extracted the mean and standard values of depression scores in the placebo group of each study. Then, we calculated the pooled effect size for escitalopram and rTMS arm separately, using Cohen's d as the measure of effect size. We found that placebo response are large for both escitalopram (Cohen's d - random-effects model - 1.48; 95%C.I. 1.26 to 1.6) and rTMS studies (0.82; 95%C.I. 0.63 to 1). Exploratory analyses show that sham response is associated with refractoriness and with the use of rTMS as an add-on therapy, but not with age, gender and sham method utilized.

**Conclusions/Significance:**

We confirmed that placebo response in MDD is large regardless of the intervention and is associated with depression refractoriness and treatment combination (add-on rTMS studies). The magnitude of the placebo response seems to be related with study population and study design rather than the intervention itself.

## Introduction

Placebo effect plays a significant role in clinical trials of major depressive disorder (MDD); in fact, two recent meta-analyses showed that the mean responder raters in the placebo group in antidepressant trials are 29.7% [1] and that drug-placebo differences might be relatively small in patients with MDD due to the large placebo response [2]. Given the importance of placebo response in MDD trials and the need to develop efficient research designs, it is critical to enhance our understanding on the placebo effects of distinct treatments such as repetitive transcranial magnetic stimulation (rTMS), a novel non-pharmacological intervention for neuropsychiatric diseases.

Several meta-analyses of rTMS clinical trials have been performed in the past ten years, initially showing negative or poor results [3], [4]; although two recent studies have demonstrated a greater efficacy of the method [5], [6]. However, taking into account the heterogeneity of rTMS trials and the lack of precise predictors of outcome, Herrmann and Ebmeier [7] proposed that non-specific contextual effects - such as the use of a new and relatively unknown technological device and the running of trials in major universities and teaching hospitals - play an important role in rTMS depression improvement. In fact, non-pharmacological treatments might have a large placebo response [8]. Furthermore, despite several meta-analyses assessing the placebo response of pharmacological trials in depression [1], [2], [9], [10] placebo response of transcranial magnetic stimulation has not been sufficiently explored. We therefore decided to assess the placebo response of such intervention and perform an exploratory comparison with a non-pharmacological intervention trough a systematic review and meta-analysis of recent clinical trials of major depression.

### Aims of the study

This study sought to ascertain the magnitude of placebo response in controlled trials of rTMS and non-pharmacological studies using escitalopram as the antidepressant drug. Our secondary aim, given the limitations for such aim, was to exploratory compare the effect sizes of placebo responses of rTMS studies and pharmacological studies. The importance of our study is contribute towards a better understanding of the placebo effects mechanisms by comparing a traditional pill-taking medical ritual to a new sham-device healing context.

## Methods

We chose escitalopram to estimate the placebo response of pharmacological treatment as several placebo-controlled trials have been recently conducted and for non-pharmacological treatment we chose rTMS as, similarly, several sham-controlled studies have also been performed recently. We performed a systematic review on all escitalopram and rTMS trials published since 2002 and subsequently performed two main analyses: for the placebo-drug response and for the sham-rTMS response. We then compared the effect size of these groups. We also performed exploratory analyses to assess predictors associated with placebo response.

We choose this time period because the first escitalopram trial was published in 2002 and we looked for concurrent rTMS and escitalopram trials to make the studies more comparable methodologically (i.e., with comparable sample sizes, diagnostic definitions, rating methods and quality of studies) and also because a meta-analysis performed in 2003 [3] stated that rTMS trials up to 2002 had been of low quality.

### Literature Search

We searched for published articles from April 2002 to April 2008 (period of 96 months) in the following databases: MEDLINE, Web of Science, Cochrane, and SCIELO. We also examined reference lists in systematic reviews and retrieved papers. To check for unpublished trials, we: (i) consulted the CRISP database and the websites clinicaltrials.gov and clinicalstudyresults.org; (ii) contacted experts; (iii) searched for conference poster abstracts; (iv) searched for studies in the monograph reference lists of Lexapro ® and; (iv) sent e-mails asking for unpublished studies to Forest Labs and to Lundbeck S/A. Our key search terms were “depression”, “escitalopram”, and “placebo” in the escitalopram arm; and “depression”, “transcranial magnetic stimulation” and “sham” in the rTMS arm.

### Selection criteria

The following inclusion criteria were adopted: (i) manuscript written in English (although there were no manuscripts in other languages); (ii) randomized, double-blinded, placebo-controlled (or sham-controlled), parallel studies on major depressive disorder; (iii) mood effects assessed by a continuous mood scale, such as Hamilton Depression Rating Scale (HDRS) or Montgomery-Asberg Depression Rating Scale (MADRS); (iv) studies that reported mean and standard deviation of the mood scales (or provided other statistical parameters that could be used to deduce this values) for the placebo group and; (v) studies published from April 2002 to April 2008.

### Data extraction

Data were extracted independently by the first author (AB) and double-checked by the second author (ML), using a structured form. The discrepancies were resolved by consensus and the corresponding author (FF) consulted if needed. The following variables were extracted: 1) mean and standard deviation values of depression rating scales at baseline and end of treatment in active (active group was used for exploratory analysis) and placebo/sham groups and; 2) demographic, clinical and treatment characteristics (e.g. number of patients, age, gender, previous use of medications, depression-resistant subjects, duration of treatment, sham procedure utilized).

When the study did not report mean and standard deviation (SD) values, we either deduced them (using statistical parameters) or contacted the corresponding author. Many escitalopram studies did not report SD final scores – in these cases, we calculated SD from standard error (SE) at end-of-treatment or from SD or SE difference changes when possible [11], [12]. In two studies, SD had been only reported in graphs and we asked for data from Forest Research Institute [13], [14]. We also received data from Forest Labs of two posters [15], [16] and for an unpublished trial mentioned in another study [13]. Two authors failed to provide the requested data [17], [18]; in these cases we had to input SD post-treatment scores based on the mean of the available SD scores of other trials, a method suggested by The Cochrane Collaboration to be applied in such cases [19]. In the rTMS arm, some trials just reported data in graphs, while others did not report SD post-treatment scores. We contacted the corresponding authors in these cases [20], [21], [22], [23]. Many rTMS trials also reported several depression scores at different times using more than one depression scale -in such cases, we extracted the data presented by the authors as the main result. Finally, we used only unadjusted rating scores in our analysis.

### Quality assessment

We looked for the following biases: (1) selection bias - adequate concealment of treatment (e.g., randomization was performed by lottery and sealed, opaque envelopes were used); (2) performance bias – if the study is single-blinded or double-blinded - for rTMS studies we checked if they were single-blinded studies with external blind raters and also if blinding of patients and physicians were assessed; (3) attrition bias – if data are adequately reported in the study, if there is evidence of intention-to-treat treatment, and if methods used to handle with missing data (e.g., last observation carried forward, complete case analysis) were reported.

### Quantitative analysis

All of our analyses were performed using STATA statistical software, version 9.0 (Statacorp, College Station, TX, USA). We initially calculated the standardized mean difference and the pooled standard deviation for each comparison –i.e. for each study we calculated the change of either placebo or sham scores (baseline minus post-treatment scores) and divided by the standard deviation of change. We used Cohen's *d* as a measure of the effect size. Then, we measured the pooled weighted effect size (weighted by the inverse variance of each study) using the random and fixed effect models. We performed the analyses of placebo response in escitalopram and rTMS trials separately and further compared the pooled effect sizes. Heterogeneity was evaluated with Chi-square test. We also performed sensitivity analysis, cumulative regression and assessed publication bias using Begg-modified funnel plot and Egger test [24] for each analysis.

Meta-regression was performed using the random-effects model and tau^2^ variance was calculated by the method of the residual maximum likelihood. We tested the following variables: age (years), gender (%females), duration of treatment (weeks), and depression response in the active groups (Cohen's *d* pooled effect size of the active groups) – treated as continuous variables; sham procedure, treatment resistant patients (defined as more than 50% of patients failed at last two antidepressant treatments); drug-free patients; and *rTMS as an add-on therapy* were treated as categorical variables. It should be underscored that we classified as “angled coil” studies that described the use of an active rTMS coil in a different angle or position when applied to the scalp; whereas “sham coil” included studies that used a non-active coil associated with a method to preserve blindness (e.g. a study [25] described that sham stimulation was performed with “an identical coil (…) but without any electronic connection. This set-up had a similar sound effect but with no stimulation…”). Three studies used a different sham approach and were not pooled together in this analysis, because either a shielded coil [26] or a special coil generating a small field [23] were used.

Also, we considered as “add-on therapy” when a drug treatment was initiated simultaneously to active or sham rTMS, i.e., patients from sham group were actually starting an active drug treatment -in fact, this is the same concept of an “accelerating” study [27].

For baseline depression, we meta-regressed using either MADRS or HDRS baseline scores in escitalopram and rTMS trials, respectively. For rTMS studies that used MADRS scores as the primary outcome, we used the values of HDRS scores reported in secondary outcomes when this was possible [26], [28], [29], [30], [31]; in four studies this was not possible [20], [32], [33], [34] and therefore we imputed missing HDRS scores regressing for other variables. Finally we assessed whether improvement in the active group was correlated with the placebo response – including this variable in our model.

## Results

**Figure 1:**
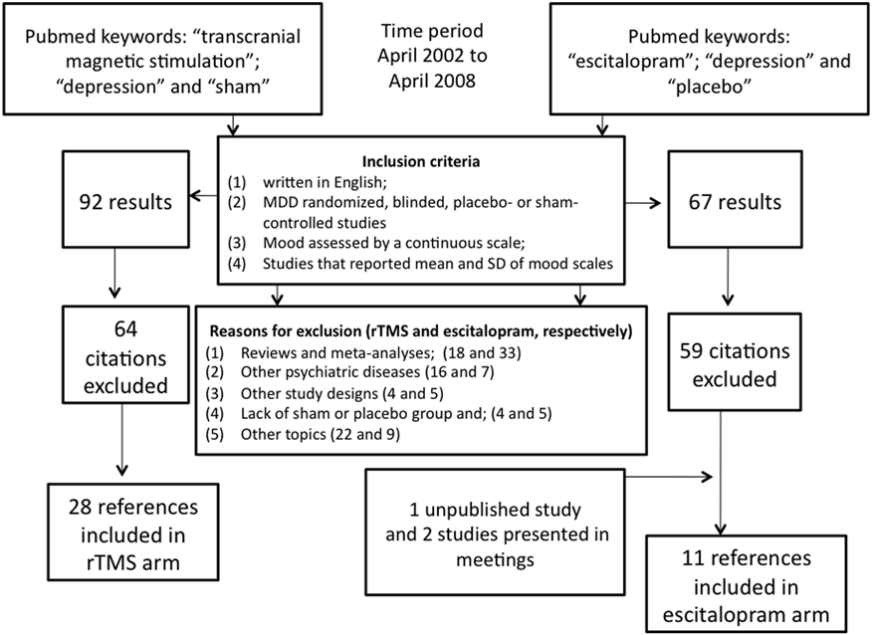
QUOROM flow chart used to identify studies for detailed analysis.

Using the keywords previously mentioned we were able to find 67 citations for escitalopram and 92 for rTMS studies. Only 12 and 29 studies met our inclusion criteria. Reasons for exclusion included: (1) reviews and meta-analyses; (2) studies that assessed other psychiatric diseases; (3) other studies designs (open-label, cross-over designs, quasi-randomized trials); (4) lack of sham or placebo group; (5) other topics. (Fig. 1)

Regarding study quality, all escitalopram studies are multi-centric, randomized (although only one study reports the allocation method), double-blinded, and performed an intention-to-treat analysis (ITT), using the last observation carrier forward (LOCF) method. The quality of the rTMS studies is heterogeneous: all studies are randomized (thirteen studies report the allocation method); and single-blinded with external evaluation but only 8 studies addressed the integrity of blinding. Eighteen studies performed an intention-to-treat analysis, while 11 performed a complete-case analysis – mostly, exploratory studies. Finally, only two rTMS studies are multicentric. The quality assessment of each study is reported in Table S1.

The clinical characteristics of the 41 studies are summarized in Table 1. Tables 2 and 3 show characteristics of each study. There were 680 patients in sham group in the 29 rTMS studies (median per study = 16, interquartile range (IQR) = 10–26), while the 12 escitalopram studies enrolled 1714 patients in the placebo group (median per study = 133, IQR = 128–153). Also, all escitalopram studies enrolled non-treatment resistant patients who were drug-free, while most patients in rTMS studies were refractory and using antidepressant drugs – in fact, in 6 studies an antidepressant drug was initiated in both active and sham groups at the beginning of the trial. Conversely, the groups were comparable regarding age (50.7 vs. 43.1 years), gender (59% vs. 61% females) and baseline HDRS (24.73 vs. 21.4) and MADRS (33.1 vs. 29.23) scores.

**Table 1:** General characteristics of the studies.

	rTMS	Escitalopram
Number of studies	29	12
Patients in active group	715	1967
Patients in sham/placebo group	680	1714
Age (mean±SD) *	50.76 (7.56)	43.1 (16.6)
Gender (%female) *	59%	61%
Refractory to Antidepressants (%)	73%	0
Concomitant antidepressant use in placebo group(%)	76%	0
Studies that used HDRS in the primary outcome	20	3
HDRS baseline scores (mean±SD) *	24.73 (4.47)	21.4 (3.20)
HDRS post-treatment scores (mean±SD) *	19.78 (3.57)	11.4 (0.3)
Studies that used MADRS in the primary outcome	9	8
MADRS baseline scores (mean±SD) *	33.1 (3.59)	29.23 (0.90)
MADRS post-treatment scores (mean±SD) *	26.2 (6.25)	17.72 (1.96)

SD = standard deviation; MADRS = Montgomery-Asberg Depression Rating Scale; HDRS = Hamilton Depression Rating Scale; rTMS = repetitive transcranial magnetic stimulation. (*) In sham/placebo group.

**Table 2:** Characteristics of each rTMS study included.

Author and Year	Patients in sham group	Age (mean)	N of female	Depression scale	Baseline sham scores	Post-tto sham scores	Add-on therapy	Treatment resistant	Concomitant AD use
Boutros [57]	9	49.5	1	HDRS	35.44	26.42	No	Yes	Yes
Hoppner [28]	9	56	6	MADRS	37.5	29	No	No	Yes
Fitzgerald [32]	20	49.15	11	MADRS	35.75	35.4	No	Yes	Yes
Loo [33]	10	54.9	6	MADRS	33.1	27	No	Yes	Yes
Herwig [21]	12	47.8	8	HDRS	23.1	14.5	No	Yes	Yes
Koerselman [58]	26	52	17	HDRS	25.9	20.2	No	No	Yes
Poulet [20]	9	N/A	4	MADRS	36.22	18.125	Yes	No	Yes
Holtzheimer [59]	7	45.4	4	HDRS	20.8	15.3	No	Yes	No
Jorge [60]	10	66.5	5	HDRS	20.8	20	No	Yes	No
Mosimann [61]	9	64.4	5	HDRS	24.5	20.4	No	Yes	Yes
Hausmann [62]	13	47	9	HDRS	33.7	20.2	Yes	N/A	Yes
Rossini (J Clin Psych)[63]	49	46.4	40	HDRS	25.1	16.8	Yes	No	Yes
Rumi [64]	24	38.9	20	HDRS	29.71	22.1	Yes	No	Yes
Rossini (Psych Res), [22]	17	56.3	11	HDRS	28.7	24.9	No	Yes	Yes
Su [65]	10	42.6	7	HDRS	22.7	19	No	Yes	Yes
Januel [66]	16	37.19	12	HDRS	22.5	16.69	No	No	No
Fitzgerald [31]	27	43.7	16	MADRS	34	30.9	No	Yes	Yes
Avery [67]	33	44.2	16	HDRS	23.5	20	No	Yes	Yes
Garcia-Toro [68]	10	47.2	7	HDRS	25.1	23.67	No	Yes	Yes
Loo [29]	19	45.7	8	MADRS	32.6	27.1	No	No	Yes
Herwig [30]	65	49	32	MADRS	27.1	16.3	Yes	No	Yes
O'Reardon [26]	146	48.7	74	MADRS	33.9	30	No	Yes	No
Anderson [34]	16	48	9	MADRS	27.7	21.9	No	Yes	Yes
Stern [69]	10	53.3	6	HDRS	27.4	26.7	No	Yes	Yes
Bortolomasi [70]	7	55.6	4	HDRS	22	19	No	Yes	Yes
Jorge1 [23]	15	66.1	8	HDRS	19.9	16.8	No	Yes	No
Jorge2 [23]	29	62.1	17	HDRS	17.6	14.8	No	Yes	No
Mogg [71]	30	52	21	HDRS	21.6	19.4	No	Yes	Yes
Bretlau [25]	23	57.8	13	HDRS	24.7	19.1	Yes	Yes	Yes

rTMS = repetitive transcranial magnetic stimulation; AD = antidepressant drug; HDRS = Hamilton Depression Rating Scale; MADRS = Montgomory-Asberg Depression Rating Scale; N/A = data not available.

**Table 3:** Characteristics of each escitalopram study included.

Author and Year	Patients in placebo group	Age (mean)	N of female	Depression scale	Baseline placebo scores	Post-tto placebo scores	Weeks of treatment	Treatment resistant
Burke [11]	119	40	71	MADRS	29.5	20.1	8	No
Wade [72]	189	40	147	MADRS	28.7	16.7	8	No
Lepola [73]	154	43	111	MADRS	28.7	16.2	8	No
Ninan [15]	153	39	99	MADRS	30.5	20.5	8	No
Rapaport † [13]	127	42.2	74	MADRS	28.8	17.5	8	No
Alexopoulos [16]	132	N/A	75	MADRS	30.7	18.4	8	No
Kasper [17]	180	75	137	MADRS	28.6	14.6	8	No
Clayton2 [12]	126	37	73	HDRS	23.3	11.4	8	No
Clayton1 [12]	130	35	81	HDRS	23.2	11.1	8	No
Wagner [74]	133	12.4	69	CDRS	56.6	36.4	8	No
Nierenberg [18]	137	42.5	49	HDRS	17.7	11.7	8	No
Bose [14]	134	68.5	79	MADRS	28.4	17.8	12	No

† Data of unpublished study. HDRS = Hamilton Depression Rating Scale; MADRS = Montgomory-Asberg Depression Rating Scale; CDRS = Children's Depression Rating Scale; N/A = data not available.

**Figure 2:**
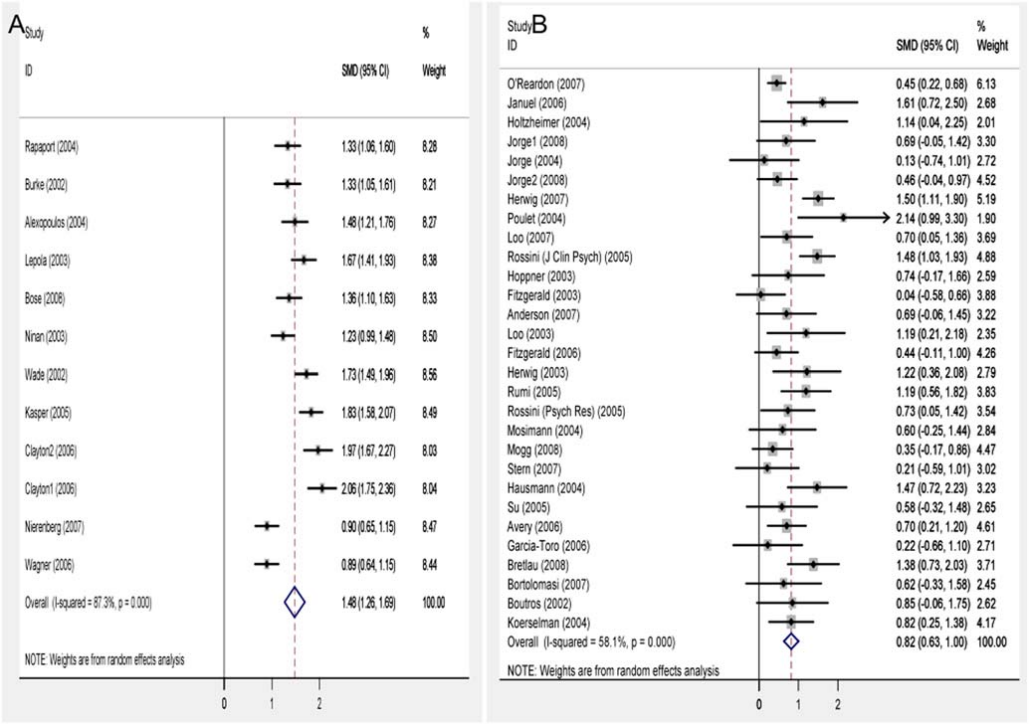
Forest plots showing placebo response in control groups of escitalopram (A) and rTMS (B) studies. Forest plots show effect sizes from the random effects model. A negative effect indicates that endpoint depression scores in control groups are higher than baseline scores. Effect sizes are Cohen's *d* (standardized mean difference), error bars represent the 95% confidence interval.

Our main results show that the pooled effect sizes for placebo response in escitalopram trials are 1.46 (95% CI 1.38 to 1.53) using the fixed-effects model and 1.48 (95% CI 1.26 to 1.69) using the random-effects model; and, for rTMS studies, the sham pooled effect size is, in the fixed-effects model, 0.77 (95% CI 0.66 to 0.88) and 0.82 (95% CI 0.63 to 1) in the random-effects model (Fig. 2). Since heterogeneity is significant in both analyses (χ2 = 86.54, p<0.001 and χ2 = 66.87, p<0.001, respectively) subsequent analyses were performed using the random-effects model. For both arms, sensitivity analysis and Begg's funnel plot show neither change in results after the exclusion of any particular study nor evidence of publication bias and systematic heterogeneity across the studies (Figures S1 and S2).

Subgroup analyses also show that the sham effect size of add-on rTMS studies (1.47, 95% CI 1.24–1.70) differ from studies not adopting such strategy (0.56, 95% CI 0.44–0.7, p<0.001), and of non-refractoriness studies (1.24, 95% CI 0.96–1.52) vs. studies with treatment-resistant patients (0.55, 95% CI 0.42 to 0.68, p<0.001) (Figure S3).

### Exploratory analysis

Simple linear regressions (table 4) show that some variables are associated with the outcome: (1) a negative association is observed for treatment resistant patients (ß coefficient = −0.69, p<0.001), meaning that refractoriness diminish placebo response; (2) a positive association (ß coefficient = 0.9, p<0.001) is observed for rTMS as add-on therapy, meaning that placebo response increases in accelerating studies and; (3) a positive association is observed for depression improvement in active groups for both escitalopram (B = 0.7, p<0.0001) and rTMS studies (B = 0.33, p = 0.002), i.e., studies showing a large depression improvement in active group also showed a large depression improvement in the control group.

**Table 4:** Meta regression results in which several variables were analyzed trough simple linear regressions.

Explanatory variables	Escitalopram			rTMS		
	d.f.	Coef. (B)	p	d.f.	Coef. (B)	p
Baseline MADRS/HDRS	6	−0.12	0.23	27	0.52	**0.04**
Depression scale (HDRS vs MADRS)	10	−0.15	0.40	27	0.05	0.79
Gender (n Female)	10	<0.001	0.09	27	<0.001	0.96
Age (years)	9	0.07	0.36	26	−0.2	0.14
Patients using ADs (Y/N)	-	-	-	27	0.21	0.36
Tto Resistant (Y/N)	(*)			26	−0.69	**<0.0001**
Week of post-tto scores	(**)			27	−0.03	0.56
Sham method (Coil angled vs. Sham coil)	-	-	-	24	−0.21	0.35
rTMS as add-on therapy (Y/N)	-	-	-	27	0.90	**<0.0001**
Active Group change (Cohen's d)	10	0.70	**<0.0001**	27	0.33	**0.002**

Coef. (B) is the regression coefficient of each regression, representing the slope of each model. Significant observations (p<0.05) are highlighted in bold. D.f. = degrees of freedom; HDRS = Hamilton Depression Rating Scale; rTMS = repetitive transcranial magnetic stimulation; AD = antidepressant drug; MADRS = Montgomery-Asberg Depression Rating Scale; MDD = Major Depressive Disorder; Y/N = yes or no. (*) There are no escitalopram studies that enrolled patients with refractory MDD. (**) All escitalopram studies except one assessed post-treatment scores at week 8.

On the contrary, baseline HDRS scores, baseline MADRS scores, depression scale utilized, gender and age are not associated with placebo response for both escitalopram and rTMS studies - except for baseline HDRS scores in rTMS studies (B = 0.52, p = 0.04), i.e., depression severity associates with a large placebo response.

Finally, variables that were associated with the outcome (p<0.1) were included in multiple linear regressions (Table 5). We observe that in models 1a (covariates: *active-rTMS treatment* and *add-on therapy*) and 1b (covariates: *HDRS baseline scores* and *add-on therapy*) only *rTMS as add-on therapy* remains associated with the outcome. Similarly, the variable *baseline HDRS scores* looses significance when meta-regressed together with *treatment resistant* (model 2b); however, in model 2a, both *depression improvement in active-rTMS group* (B = 0.2, p = 0.02) and *treatment resistant* (B = −0.57, p<0.0001) associates with placebo response. In model 3 all mentioned variables are regressed together; results show that only *rTMS as add-on therapy* still positively associates with the outcome (B = 0.53, p = 0.02), although there is still a trend for a negative association for *treatment resistant* variable (B = −0.31, p = 0.08), meaning that such variables still modify placebo response even when controlled by other significant variables.

**Table 5:** Exploratory regression models for rTMS studies in which significant results (obtained from simple linear regressions) were forced into several models.

Regression model	Variable	D.f.	Coef (B)	p
**Model 1a**	Active rTMS Group improvement	26	0.12	0.14
	rTMS as add-on Therapy		0.77	**<0.0001**
**Model 1b**	Baseline HDRS scores	26	0.15	0.45
	rTMS as add-on Therapy		0.86	**<0.0001**
**Model 2a**	Treatment resistant	26	−0.57	**<0.0001**
	Active rTMS Group improvement		0.20	**0.02**
**Model 2b**	Baseline HDRS scores	25	0.03	0.25
	Treatment resistant		−0.49	**<0.0001**
**Model 3**	Baseline HDRS scores	23	0.01	0.86
	Treatment resistant		−0.31	0.08
	Active rTMS Group improvement		0.11	0.21
	rTMS as add-on Therapy		0.53	**0.02**

Coef. (B) is the regression coefficient of each regression, representing the slope of each model. Significant observations (p<0.05) are highlighted in bold. D.f. = degrees of freedom; HDRS = Hamilton Depression Rating Scale; rTMS = repetitive transcranial magnetic stimulation.

## Discussion

This meta-analysis includes data from 12 escitalopram and 29 rTMS trials, assessing 2394 subjects in placebo/sham groups. Our main result shows that placebo response is large in major depression trials, regardless of the placebo method. Exploratory analyses found that patients with severe depression and with treatment-resistant depression present a lower placebo response; while in trials that rTMS is initiated concomitantly with an antidepressant drug, the placebo response is larger.

The main finding of our study is that both placebo interventions are associated with a large effect size in major depressive disorder, which is in line with previous studies: Walsh et al. [1] reviewed 75 depression trials and concluded that placebo response is substantial and increasing over years; Stein et al. [35] in a pooled analysis of five escitalopram trials showed that placebo response ranged from 31.6% to 45.9%; and Kirsch et al [2], reviewing 35 published and unpublished trials, showed that placebo response ranged from 0.7 to 1.1 Cohen's *d*. Therefore our study confirms that placebo response is substantial in pharmacological and non-pharmacological trials in major depression.

Another finding of our study is that placebo-drug response appears to be larger than sham-rTMS response – even controlling for treatment refractoriness. Even considering that the small difference might not be meaningful, this finding is contrary to conventional wisdom that sham devices would have a higher placebo response than placebo pills [8], [36]. In fact, a non-pill intervention showed increased response than a placebo pill in a prospective sham device vs. inert pill trial [37] and in a meta-analysis comparing subcutaneous placebo with oral placebo from acute migraine [38]. Our finding does however agree with a smaller acute care study that found no difference between parenteral medication and oral medication [39]. These differences could be related to the concept that placebo response is very heterogeneous and influenced by many variables. In our study, this finding might be explained by several factors:

1. Study populations are different: 73% of rTMS trials enrolled refractory MDD patients; whereas no escitalopram trials enrolled refractory patients – in fact, STAR*D (Sequenced Treatment Alternatives to Relieve Depression) trial shows that remission rates decay at each time an antidepressant drug fail, being only 13% for refractory patients – i.e. patients who failed to remit after two trials [40]; and, since antidepressant drug effect is partially composed by a non-specific, placebo effect, placebo response might also decay in refractory patients. Along with these lines, low placebo responses were reported in a recent rTMS meta-analysis that addressed treatment-resistant patients [41] as well as two drug meta-analyses using lithium [42] and atypical antipsychotics [43].
2. Study designs are different: although escitalopram and rTMS trials present comparable quality, they mainly differ in blinding quality, as adequate blinding is more difficult to obtain in non-pharmacological interventions [44]. The rTMS trials assessed used an approach in which patients and raters were blinded to the treatment group allocated; however, it is possible that rTMS appliers unconsciously behave different when applying real and sham stimulation as well as that patients discover in which intervention they were allocated. Unsuccessful blinding biases the results as expectation effects and intervention confidence will be lost [45], [46], [47], [48], therefore diminishing placebo response. Also, it is possible that study design influences outcome, since Woods et al. [49] showed that, in schizophrenia controlled-trials, improvement was larger in trials having no placebo arm; and Trivedi et al. [50] showed that response raters were different in depression controlled-trials regarding using or not a placebo run-in phase.
3. Study sites and approaches are different: whereas drug trials are conducted along 8 weeks, with weekly returns, rTMS trials are conducted in 2 to 4 weeks – therefore longer exposure might be associated with a larger placebo response. On the other hand, rTMS treatment is associated with an intensive 10-day treatment (as opposed to weekly or bi-weekly interaction in drug trials) and this could potentiate placebo response in the rTMS trials.

Our results show that sham-response is smaller in trials that rTMS is not used as add-on therapy (0.56 vs. 1.24), suggesting that such device might not be associated with a large placebo effect, a finding that was also observed in meta-analyses of Parkinson's disease [51] and of refractory MDD [41]. Also, add-on rTMS trials improve response in placebo arm even when controlled for other variables, which could point to a synergistic effect between sham-rTMS and the drug, since there is no association between placebo response and previous use of antidepressant drugs. Finally, sham method (sham coil vs. angled coil) does not change placebo response – perhaps because both approaches, in fact, do not guarantee blinding.

### Limitations

There was significant between-study heterogeneity in our meta-analysis, suggesting that the variation of effect size estimates in the studies were more than expected by chance. To address this limitation, we (1) used a random-effects model, which is a more conservative pooled analysis that take into account the between-study heterogeneity; (2) performed sensitivity analyses, to address whether the exclusion of an study could affect the pooled effect size; (3) assessed the quality of each study, looking for potential biases; and (4) checked for publication biases using Begg's funnel plot.

Another limitation is that, for pharmacological studies, we only included escitalopram studies; consequently, it is possible that the placebo response of other drugs is different. However, our study is in line with previous meta-analyses that showed similar placebo responses in major depression studies [1], [52], assessed a significant number (1714) of patients and included unpublished studies; therefore this hypothesis is less likely.

Finally, it should be emphasized that the secondary analyses performed are exploratory and might be underpowered; in fact, since ten linear regressions have been performed in each pooled analysis, there is a 50% probability of observing one positive association merely due to chance.

### Clinical implications

Because we addressed the influence of several variables in sham-response, our results have some implications for future rTMS trial designs, such as: (1) sham device method is not associated with placebo response; therefore this factor seems less relevant than currently considered by the researchers in this field [53]; (2) age and gender are probably not related with placebo response - although age seems to be related to depression response in some studies [54], [55]; (3) refractoriness is associated with a lower placebo response – and, in fact, a lower depression response [41], [54] [56]; perhaps indicating that such patients are very unresponsive to any intervention at all and therefore rTMS studies should focus on non-refractory patients or, on the contrary, the positive results of rTMS trials might be due to a lower placebo response that increases active-sham difference – therefore, future rTMS trials should quantify the degree of refractoriness of each patient, and; (4) placebo response is high in add-on rTMS trials – this could indicate there is a synergistic effect with the drug and, therefore, future trials could use a two-way factorial design (i.e., sham vs. real-rTMS and placebo vs. active drug) to address the relationship among rTMS and drug interventions.

Our study also stresses the heterogeneity of placebo response in different contexts and interventions; therefore, the lower placebo response observed in sham trials could be explored by using a qualitative approach to understand patient's expectancies regarding rTMS intervention or, perhaps, by a sham-device vs. inert pill trial, in the same fashion of a prior placebo study [37].

### Final remarks

In summary, our study shows that placebo response in rTMS and escitalopram trials is large and appears to be lower for rTMS trials. The sham response is negatively associated with refractoriness and positively associated with rTMS add-on studies; whereas sham method utilized, age and gender are not associated with a greater sham response. It is possible that design issues such as the lack of adequate blinding associate with lower placebo responses; however, we cannot measure in which extent such difference is explained by other cultural factors, as pill-taking healing is a mainstream medical ritual, while sham devices are not. The sham response of rTMS significantly varies among studies and can influence the results of a clinical trial as it will determine the effect size of a given sham-controlled trial, therefore, further studies are needed to explore its effects as to design appropriate sham-controlled randomized clinical trials.

## Supporting Information

Table S1 The file contains the quality assessment of all the studies included.
(0.57 MB PPT)

Figure S1 (A) shows the sensitivity analysis, assessing the individual influence of a particular study by showing the resulting effect size and 95% confidence interval (CI) after its exclusion. (B) shows the funnel plot of the effect sizes (Cohen's d) according to their standard errors. Cohen's d is the standardized mean difference, error bars represent the 95% CI.
(3.00 MB TIF)

Figure S2 (A) shows the sensitivity analysis, assessing the individual influence of a particular study by showing the resulting effect size and 95% confidence interval (CI) after its exclusion. (B) shows the funnel plot of the effect sizes (Cohen's d) according to their standard errors. Cohen's d is the standardized mean difference, error bars represent the 95% CI.
(3.00 MB TIF)

Figure S3 (A) shows the influence of the variable add-on rTMS in the pooled analysis of the studies, by pooling together only studies in which this variable is present (top) or absent (bottom) and thereby comparing the resulting effect sizes (Cohen's d, standardized mean difference). (B) shows the influence of the variable treatment-resistant depression, when it is present (top) or absent (bottom) in the resulting effect sizes.
(3.00 MB TIF)
