# Supplementary material for: Placebo Response of Non-Pharmacological and Pharmacological Trials in Major Depression: A Systematic Review and Meta-Analysis
Source: PLoS One. 2009 Mar 18;4(3):e4824. doi: 10.1371/journal.pone.0004824 (PMC2653635; doi:10.1371/journal.pone.0004824)
Supplement: Table S1 — The file contains the quality assessment of all the studies included. (0.57 MB PPT) [file pone.0004824.s001.ppt]

## Slide 1
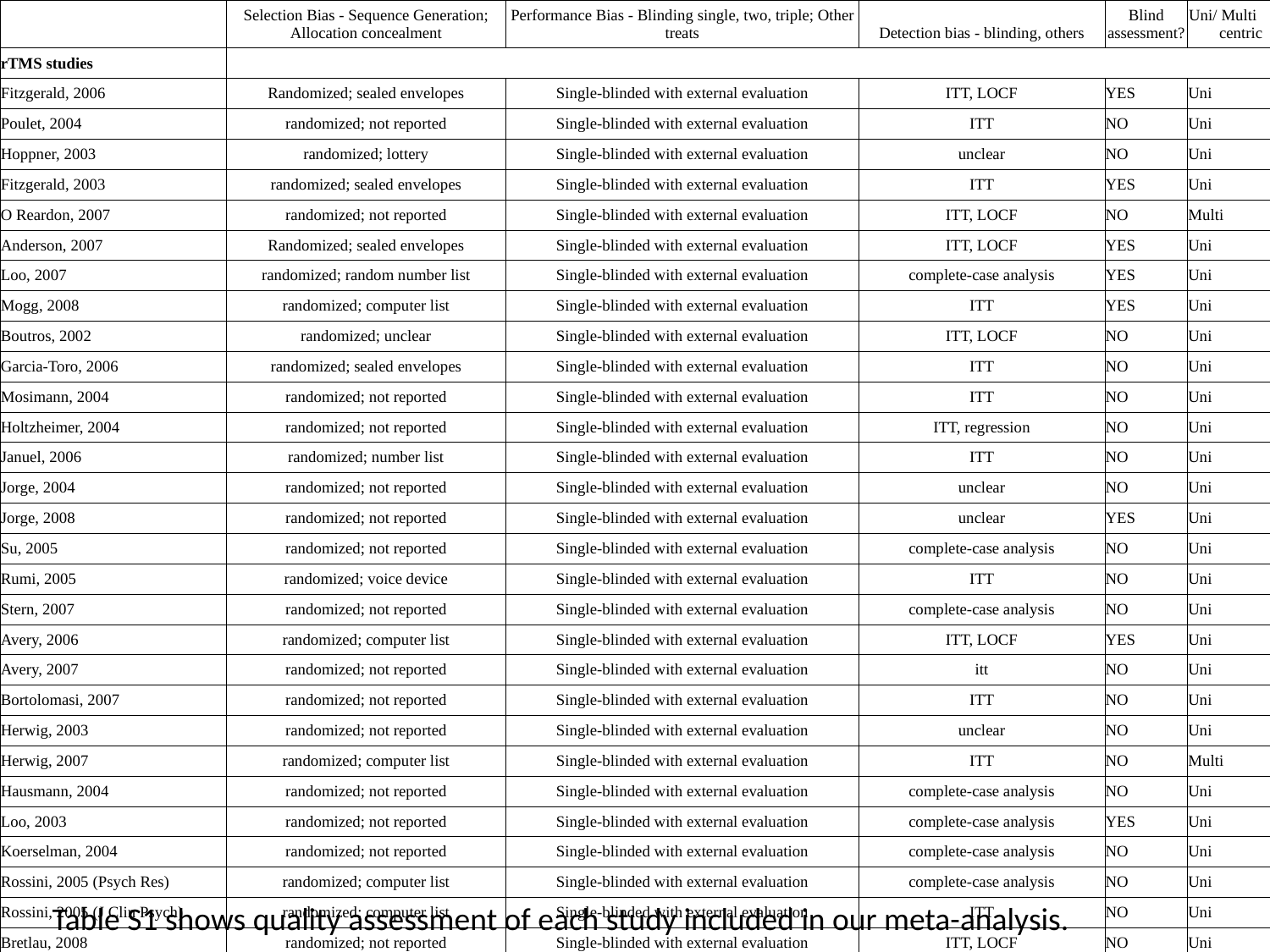

| | Selection Bias - Sequence Generation; Allocation concealment | Performance Bias - Blinding single, two, triple; Other treats | Detection bias - blinding, others | Blind assessment? | Uni/ Multi centric |
| --- | --- | --- | --- | --- | --- |
| rTMS studies | | | | | |
| Fitzgerald, 2006 | Randomized; sealed envelopes | Single-blinded with external evaluation | ITT, LOCF | YES | Uni |
| Poulet, 2004 | randomized; not reported | Single-blinded with external evaluation | ITT | NO | Uni |
| Hoppner, 2003 | randomized; lottery | Single-blinded with external evaluation | unclear | NO | Uni |
| Fitzgerald, 2003 | randomized; sealed envelopes | Single-blinded with external evaluation | ITT | YES | Uni |
| O Reardon, 2007 | randomized; not reported | Single-blinded with external evaluation | ITT, LOCF | NO | Multi |
| Anderson, 2007 | Randomized; sealed envelopes | Single-blinded with external evaluation | ITT, LOCF | YES | Uni |
| Loo, 2007 | randomized; random number list | Single-blinded with external evaluation | complete-case analysis | YES | Uni |
| Mogg, 2008 | randomized; computer list | Single-blinded with external evaluation | ITT | YES | Uni |
| Boutros, 2002 | randomized; unclear | Single-blinded with external evaluation | ITT, LOCF | NO | Uni |
| Garcia-Toro, 2006 | randomized; sealed envelopes | Single-blinded with external evaluation | ITT | NO | Uni |
| Mosimann, 2004 | randomized; not reported | Single-blinded with external evaluation | ITT | NO | Uni |
| Holtzheimer, 2004 | randomized; not reported | Single-blinded with external evaluation | ITT, regression | NO | Uni |
| Januel, 2006 | randomized; number list | Single-blinded with external evaluation | ITT | NO | Uni |
| Jorge, 2004 | randomized; not reported | Single-blinded with external evaluation | unclear | NO | Uni |
| Jorge, 2008 | randomized; not reported | Single-blinded with external evaluation | unclear | YES | Uni |
| Su, 2005 | randomized; not reported | Single-blinded with external evaluation | complete-case analysis | NO | Uni |
| Rumi, 2005 | randomized; voice device | Single-blinded with external evaluation | ITT | NO | Uni |
| Stern, 2007 | randomized; not reported | Single-blinded with external evaluation | complete-case analysis | NO | Uni |
| Avery, 2006 | randomized; computer list | Single-blinded with external evaluation | ITT, LOCF | YES | Uni |
| Avery, 2007 | randomized; not reported | Single-blinded with external evaluation | itt | NO | Uni |
| Bortolomasi, 2007 | randomized; not reported | Single-blinded with external evaluation | ITT | NO | Uni |
| Herwig, 2003 | randomized; not reported | Single-blinded with external evaluation | unclear | NO | Uni |
| Herwig, 2007 | randomized; computer list | Single-blinded with external evaluation | ITT | NO | Multi |
| Hausmann, 2004 | randomized; not reported | Single-blinded with external evaluation | complete-case analysis | NO | Uni |
| Loo, 2003 | randomized; not reported | Single-blinded with external evaluation | complete-case analysis | YES | Uni |
| Koerselman, 2004 | randomized; not reported | Single-blinded with external evaluation | complete-case analysis | NO | Uni |
| Rossini, 2005 (Psych Res) | randomized; computer list | Single-blinded with external evaluation | complete-case analysis | NO | Uni |
| Rossini, 2005 (J Clin Psych) | randomized; computer list | Single-blinded with external evaluation | ITT | NO | Uni |
| Bretlau, 2008 | randomized; not reported | Single-blinded with external evaluation | ITT, LOCF | NO | Uni |
| Escitalopram studies | | | | | |
| Rapaport, 2004 | randomized; not reported | Double-blinded | ITT, LOCF | | Multi |
| Burke, 2002 | randomized; not reported | Double-blinded | ITT, LOCF | | Multi |
| Alexopoulos, 2004 | randomized; not reported | Double-blinded | ITT, LOCF | | Multi |
| Lepola, 2003 | randomized; not reported | Double-blinded | ITT, LOCF | | Multi |
| Bose, 2008 | randomized; not reported | Double-blinded | ITT, LOCF | | Multi |
| Nina, 2003 | randomized; not reported | Double-blinded | ITT, LOCF | | Multi |
| Wade, 2002 | randomized; not reported | Double-blinded | ITT, LOCF | | Multi |
| Kasper, 2005 | randomized; not reported | Double-blinded | ITT, LOCF | | Multi |
| Clayton, 2006 | randomized; not reported | Double-blinded | ITT, LOCF | | Multi |
| Nierenberg, 2007 | randomized; not reported | Double-blinded | ITT, LOCF | | Multi |
| Wagner, 2006 | randomized; computer list | Double-blinded | ITT, LOCF | | Multi |
Table S1 shows quality assessment of each study included in our meta-analysis.
