# Supplementary figures and images for: Placebo Response of Non-Pharmacological and Pharmacological Trials in Major Depression: A Systematic Review and Meta-Analysis
Source: PLoS One. 2009 Mar 18;4(3):e4824. doi: 10.1371/journal.pone.0004824 (PMC2653635; doi:10.1371/journal.pone.0004824)

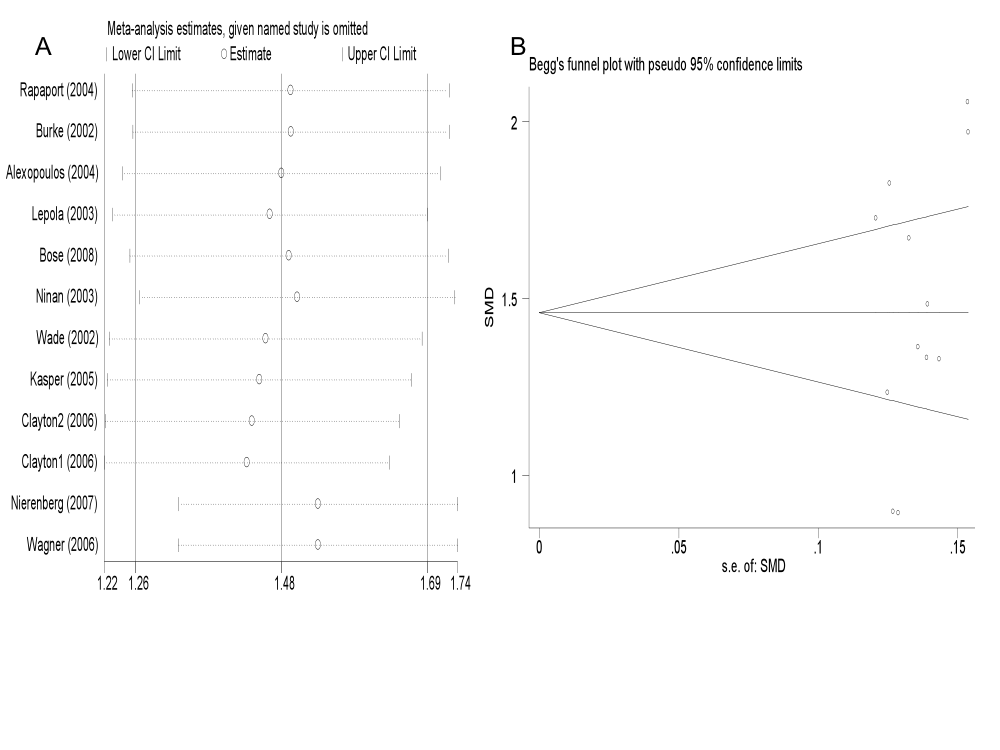

Supplement: Figure S1 — (A) shows the sensitivity analysis, assessing the individual influence of a particular study by showing the resulting effect size and 95% confidence interval (CI) after its exclusion. (B) shows the funnel plot of the effect sizes (Cohen's d) according to their standard errors. Cohen's d is the standardized mean difference, error bars represent the 95% CI. (3.00 MB TIF) [file pone.0004824.s002.tif]

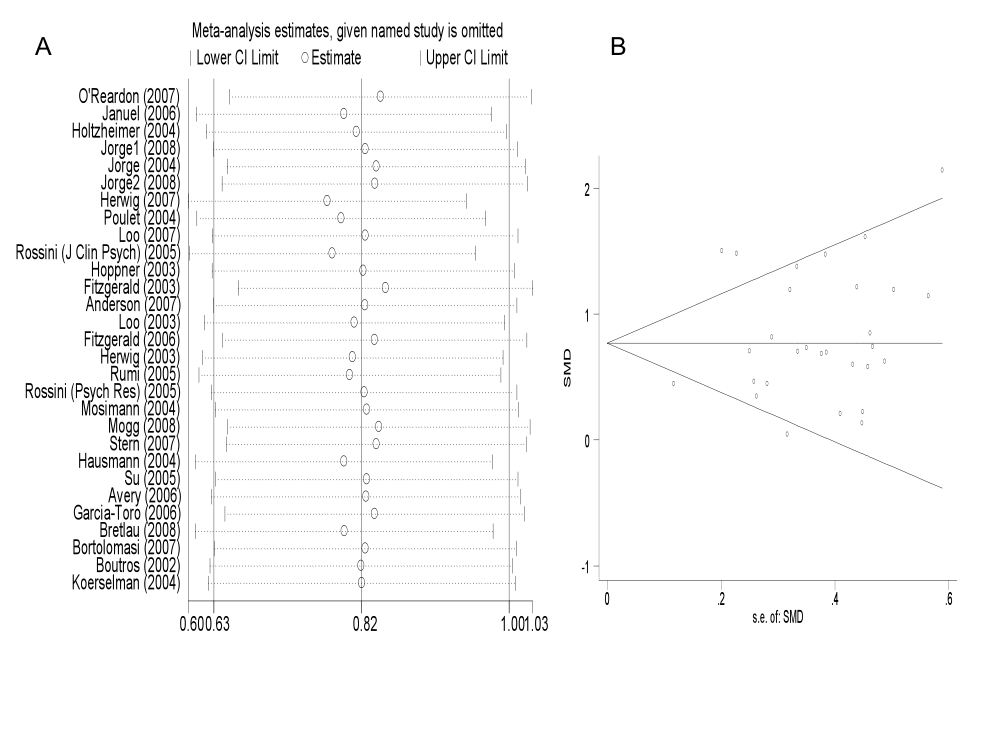

Supplement: Figure S2 — (A) shows the sensitivity analysis, assessing the individual influence of a particular study by showing the resulting effect size and 95% confidence interval (CI) after its exclusion. (B) shows the funnel plot of the effect sizes (Cohen's d) according to their standard errors. Cohen's d is the standardized mean difference, error bars represent the 95% CI. (3.00 MB TIF) [file pone.0004824.s003.tif]

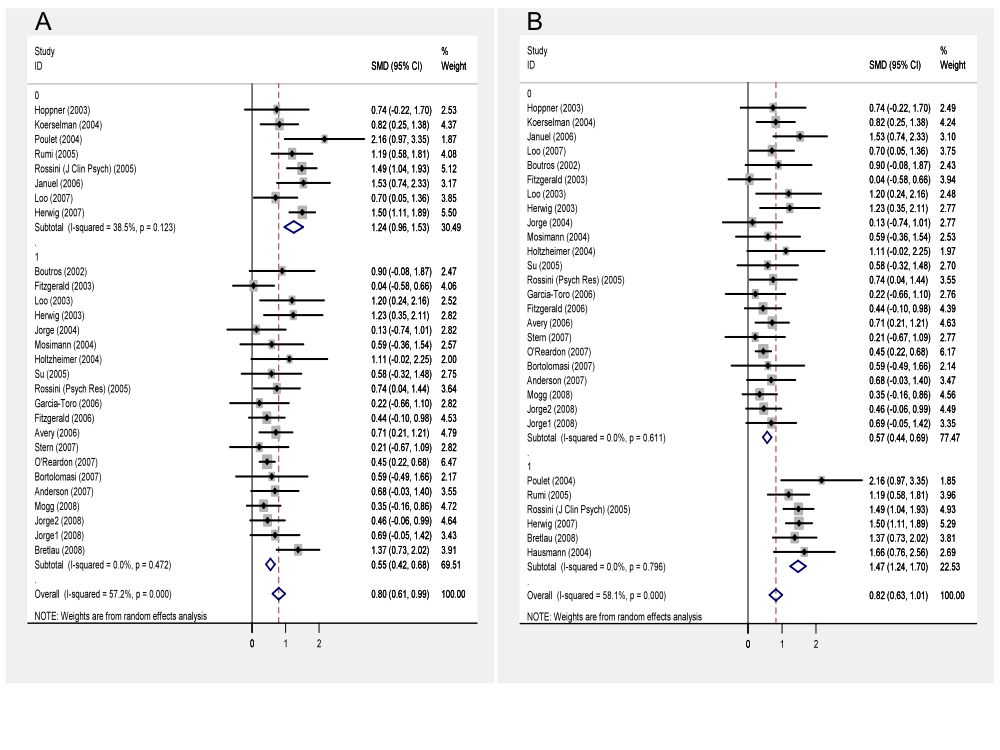

Supplement: Figure S3 — (A) shows the influence of the variable add-on rTMS in the pooled analysis of the studies, by pooling together only studies in which this variable is present (top) or absent (bottom) and thereby comparing the resulting effect sizes (Cohen's d, standardized mean difference). (B) shows the influence of the variable treatment-resistant depression, when it is present (top) or absent (bottom) in the resulting effect sizes. (3.00 MB TIF) [file pone.0004824.s004.tif]
